# Does the method matter? Direct fick vs thermodilution in the haemodynamic assessment of pulmonary hypertension

**DOI:** 10.1093/eschf/xvag093

**Published:** 2026-03-27

**Authors:** Léon Genecand, Athénaïs Boucly, Olivier Sitbon, David Montani

**Affiliations:** Department of Respiratory and Intensive Care Medicine (FHU André Cournand, ERN-LUNG), Université Paris–Saclay, Inserm UMR_S1358 (HPPIT), Hôpital Bicêtre (AP-HP), 78 Rue du Général Leclerc, Le Kremlin Bicêtre 94270, France; Faculté de Médecine, Université de Genève, Genève, Suisse; Department of Respiratory and Intensive Care Medicine (FHU André Cournand, ERN-LUNG), Université Paris–Saclay, Inserm UMR_S1358 (HPPIT), Hôpital Bicêtre (AP-HP), 78 Rue du Général Leclerc, Le Kremlin Bicêtre 94270, France; Department of Respiratory and Intensive Care Medicine (FHU André Cournand, ERN-LUNG), Université Paris–Saclay, Inserm UMR_S1358 (HPPIT), Hôpital Bicêtre (AP-HP), 78 Rue du Général Leclerc, Le Kremlin Bicêtre 94270, France; Department of Respiratory and Intensive Care Medicine (FHU André Cournand, ERN-LUNG), Université Paris–Saclay, Inserm UMR_S1358 (HPPIT), Hôpital Bicêtre (AP-HP), 78 Rue du Général Leclerc, Le Kremlin Bicêtre 94270, France

**Keywords:** Pulmonary hypertension, Thermodilution, Direct fick, Agreement

Dear editor,

We thank Melin *et al*.^1^ for their important work. Collecting more than 852 simultaneous thermodilution (TD) and direct Fick (DF) cardiac output (CO) measurements represents a substantial effort and provides important information for the field. Because DF is technically demanding and time-consuming, TD has largely replaced DF in clinical practice.^2^ However, several studies have revealed the imprecision of TD compared with DF.^3^ More generally, CO measurements, regardless of the method used, whether invasive or non-invasive show substantial imprecision reflected by wide limits of agreement (LoA) in the range of ±2 to 3 L/min across most measurement methods.^3^ Appropriately, Melin *et al*.^1^ questioned the potential impact of using DF instead of TD to measure CO on pulmonary hypertension (PH) classification. We similarly estimated this error using mathematical modelling based on the imprecision reported in the literature between DF and TD and observed results comparable with a diagnostic error of 6% across 1142 consecutive RHC,^4^ which corresponds closely to the 94% diagnostic accuracy reported by Melin *et al*.^1^ We believe that several aspects of the results warrant further discussion.

First, Melin *et al*.^1^ reported a 15% over diagnosis of a precapillary component defined by a pulmonary vascular resistance (PVR) >2 WU, when using TD. This observation should be interpreted in the light of the low mean PVR of 2.5 WU reported in their cohort. The risk of misclassification intrinsically depends on the absolute level of PVR and is highest in patients whose measurements lie close to the diagnostic threshold.^4^ These patients typically belong to group 2 or 3 PH. Such misclassification is unlikely to have major clinical consequences, as these patients are most often managed similarly regardless of mild or moderate PH, with treatment primarily targeting the underlying cardiac or pulmonary disease rather than pulmonary arterial hypertension (PAH)-approved drugs.^2^ In contrast, the risk of misclassification in patients with group 1 PAH, who typically present with substantially higher PVR values, is expected to be very low. This point is critical, because these patients benefit from PAH-specific therapies and a diagnostic misclassification could have significant clinical implications. In this context, we believe that the clinical impact of the results of Melin *et al*.^1^ could be further strengthened by providing an analysis of misclassification rates stratified according to clinical PH groups.

Second, the authors concluded that TD overestimates CO in low-flow state and underestimates CO in normal-flow states. We believe that this assumption relies on relatively limited evidence. It is possible that the observed biases of −0.38 L/min in low CO states and +0.22 L/min in normal CO states may simply reflect random variation; this issue could be assessed if the authors provided the 95% CI of the absolute bias.^5^ In high-output states, the bias appears larger (−1.78 L/min), but this finding should be interpreted cautiously given the small number of measurements and the presence of five extreme outliers showing a difference between TD and DF of −6 L/min, which raises concerns regarding the reliability of either DF or TD in these particular cases.

We think that performing DF, which requires additional time and resources, should be considered in rare situations where a meaningful impact on management is anticipated based on changes in CO or PVR. In the field of PH, such situations may include RHC performed within screening programmes, such as in patients with *BMPR2* mutations or systemic sclerosis, as well as selected patients with chronic thromboembolic disease and relatively preserved resting haemodynamic. In these populations, PVR values may be only mildly elevated, and measurement imprecision may meaningfully influence diagnostic classification and management decision-making.

In conclusion, the results of Melin *et al*.^1^ highlight the inherent limitations of CO measurement and their potential impact on haemodynamic classification. These findings underscore that excessive reliance on rigid haemodynamic cut-off values for clinical decision-making is likely inappropriate. Careful clinical assessment and thorough evaluation of risk factors before RHC are probably the most important elements for minimizing diagnostic misclassification. Haemodynamic measurements should therefore always be interpreted within the broader clinical context.

## Declarations

### Data Availability

No data were generated or analysed for this manuscript.
